# Respecting relational agency in the context of vulnerability: What can research ethics learn from the social sciences?

**DOI:** 10.1111/bioe.13139

**Published:** 2023-01-29

**Authors:** Jennifer Roest, Busisiwe Nkosi, Janet Seeley, Sassy Molyneux, Maureen Kelley

**Affiliations:** ^1^ Nuffield Department of Population Health, Ethox Centre and Wellcome Centre for Ethics & Humanities University of Oxford Oxford UK; ^2^ Africa Health Research Institute KwaZulu‐Natal South Africa; ^3^ School of Law University of KwaZulu‐Natal Durban South Africa; ^4^ Department of Global Health and Development London School of Hygiene and Tropical Medicine London UK; ^5^ School of Nursing and Public Health University of KwaZulu‐Natal Durban South Africa; ^6^ Department of Tropical Medicine University of Oxford and KEMRI Wellcome Trust Research Programme Kilifi Kenya

**Keywords:** agency, autonomy, relational, research ethics, vulnerability

## Abstract

Despite advances in theory, often driven by feminist ethicists, research ethics struggles in practice to adequately account for and respond to the agency and autonomy of people considered vulnerable in the research context. We argue that shifts within feminist research ethics scholarship to better characterise and respond to autonomy and agency can be bolstered by further grounding in discourses from the social sciences, in work that confirms the complex nature of human agency in contexts of structural and other sources of vulnerability. We discuss some of the core concepts and critiques emerging from the literature on women and children's agency in under‐resourced settings, highlighting calls to move from individualistic to relational models of agency, and to recognise the ambiguous, value‐laden, and heterogeneous nature of the concept. We then draw out what these conceptual shifts might mean for research ethics obligations and guidance, illustrating our analysis using a case vignette based on research ethics work conducted in South Africa. We conclude that if research practices are to be supportive of agency, it will be crucial to scrutinise the moral judgements which underpin accounts of agency, derive more situated definitions of and responses to agency, and enable people and participants to influence these based on their own experiences and self‐perceptions.

## 1 INTRODUCTION

In recent years, bioethics and research ethics (alongside many other disciplines) have taken their own, ‘relational turn’ in terms of theory and academic discourse.^1^ Feminist theorists such as Mackenzie, Luna, Rogers, and Dodds have argued that relational accounts of vulnerability reveal obligations beyond protection from harm, including the promotion of autonomy and agency.^2^ Relational approaches understand identities, circumstances, and opportunities to be produced and shaped through interaction with social structures and processes.^3^ In this way, different kinds of vulnerability, autonomy, and agency, are understood to be socially constructed.^4^ Despite these theoretical developments, ethical guidance, and practice regarding research with people commonly considered vulnerable to harm in the research context—such as young people living in contexts of poverty—remain dominated by accounts of vulnerability that essentialise and homogenise vulnerability, and which fail to adequately recognise and respond to autonomy and agency. We argue in this paper that greater implementation of feminist relational approaches is needed within everyday research ethics practice and practical ethical guidance for research with vulnerable populations. Towards this end, we suggest that the practical ethical significance of these conceptual ethics critiques can be bolstered by the incorporation of important work on women's and children's agency developed in gender and development and childhood studies. This work confirms the complexity of human agency in contexts of structural and other forms of vulnerability, offering the potential to more accurately inform our ethical obligations.

While the concepts ‘agency’ and ‘autonomy’ are distinct—autonomy often understood as self‐determination, self‐reflection, and self‐rule, and agency as the capacity to do or act—it is agency that frequently dominates discussion within gender and development and childhood studies, with autonomy incorporated into broader conceptions of agency.^5^ In this paper, we use developments from these discourses on children and women's agency to support and build upon efforts in feminist research ethics to better characterise and respond to issues of vulnerability, autonomy, and agency in international research contexts. First, we consider how research ethics has historically struggled to account for the agency and autonomy of those considered vulnerable—there have been many advances from feminist theorists, but there are still critical gaps. We illustrate how these issues can play out in current research practice, using a vignette based on research ethics work in South Africa. We then discuss key debates in the social sciences on children and women's agency in under‐resourced settings, revisiting the vignette to draw attention to what these conceptual shifts might mean for research ethics practice and guidance, both in terms of lending weight to relational accounts already advocated for by feminist theorists, as well as adding nuance to how agency is understood and responded to in research ethics guidance and practice. We then draw out key implications for research ethics responsibilities.

## 2 AUTONOMY, AGENCY AND VULNERABILITY IN RESEARCH ETHICS: AN OVERVIEW AND ILLUSTRATION

Historically research ethics has lacked explicit acknowledgement of traits that attend vulnerability in people's lives, such as agency and resilience, risking that research practices create harm through being overly paternalistic, disempowering, or coercive.^6^ There have been important shifts in recent years, driven by feminist theorists such as Luna, Mackenzie, Dodds, Rogers, and Lange, towards recognising context‐specificity and variation in vulnerability, and the importance of autonomy and agency for ensuring ethical research practice.^7^ Many now consider the harm principle insufficient, warning that research may exacerbate vulnerability if it fails to foster participants' agency and autonomy.^8^ Lange et al. argue, for instance, that researchers have a duty to promote the autonomy and agency of vulnerable participants for instrumental reasons, since individuals with more autonomy are likely more resilient, as well as for their own sake, since autonomy and agency are, ‘intrinsically valuable aspect[s] of human life.’^9^ Consequently, there has been greater emphasis on the ethical and scientific benefits of participant involvement in research design and implementation, and increased promotion of the voices, rights, and capacity for participation of those considered vulnerable.^10^ This shift has been accompanied by cautions against tokenistic, inappropriate, and inexpert uses of participatory approaches, with longstanding calls to ensure equitable and genuine forms of engagement.^11^

Despite advances in theory, often driven by feminist ethicists, research ethics practice and guidance still struggle to adequately account for and respond to the agency and autonomy of people deemed vulnerable in important ways. First, though several scholars emphasise the importance of relational approaches within research ethics, guidance still largely reifies more individualistic understandings of autonomy. Luna and others have argued forcefully against relying on these types of accounts, as they can perpetuate stigmatising stereotypes and prevent certain (mostly marginalised) groups from having their agency and autonomy recognised and respected.^12^ However, in their analysis of national and international research ethics policies and guidelines, Bracken‐Roche et al. found that, despite the scholarly support for relational accounts, all the policies and guidelines they reviewed still conveyed vulnerability as a personal characteristic.^13^ They highlighted that the over‐focus on individual participants in guidance can obfuscate disempowering aspects of broader research environments. We note in addition that this can also hide more empowering aspects, and there is currently no guidance for researchers on how best to evaluate the presence or lack of social support for those who might benefit from and be able to engage meaningfully in research but are considered too vulnerable to participate. This challenge is seen most clearly in research with unaccompanied children.^14^ The challenges of under‐inclusion are well appreciated, but the converse is also true: participants may be recruited and give consent but risk harm through participation, lacking adequate social support to help with literacy, language barriers, and other power imbalances which make it difficult to report side‐effects or hidden burdens of research participation or to feel free to withdraw without losing the support of researchers. Researchers have little guidance on how to strike the right balance between overprotection, avoiding potentially harmful or exploitative inclusion, and supporting and engaging the varied levels of agency amongst such participants.

Where interdependence and relational aspects are acknowledged within guidance on research with vulnerable populations, attention often remains focused on power dynamics between clinician/researcher and participant.^15^ This fails to capture other important forms of interconnection and dependence between people, institutions, structures, and processes. Indeed, the manner in which people's experiences of vulnerability, autonomy, and agency are derived from and reproduced through their interactions with structural factors (political, economic, historic, and socio‐cultural) is particularly under‐accounted for within current research ethics guidance, as well as within the regulatory structures through which they are produced.^16^

A further challenge stemming from individualistic accounts of agency is that they can perpetuate Global North notions of independent, autonomous actors, becoming easily embedded in the very fabric of research, where tools for data collection, analysis, measuring progress and change, are based on individualistic scales, indicators, and frameworks. Simply translating tools and concepts into different languages for different contexts overlooks the possibility that something different is being observed.

A second area where research ethics practice and guidance still struggles with regard to agency is that, although feminist ethicists have long called for additional research obligations to foster agency, less attention has been paid to the meaning of agency (compared to autonomy) in this context. The definition frequently remains vague and lacking in clarity, referred to broadly as the ability to choose to do things or to act. However, agency can be defined in multiple ways, including individualistically, juxtaposed with structure, or relationally. There are significant implications for research ethics guidance and practice in how agency is defined and it is important that definitions underpinning responses to vulnerability do not go unstated.^17^ Whilst feminist theorists have done much to explore and advocate for the concept of relational autonomy, we argue here that these accounts could be strengthened (both in theory and in practice) by understandings of women and children's agency developed in childhood studies and gender and development discourse. For instance, whilst feminist critiques of population‐based accounts of vulnerability in research ethics have highlighted the importance of recognising diversity and context‐specificity in experiences of vulnerability, there has been a tendency to overlook diversity in experiences of agency across different situations, people, and contexts. This homogenisation can engender inappropriately uniform responses to both vulnerability and agency, and indeed most research ethics guidelines remain too general and high level to assist on how best to protect and promote the agency of specific persons in specific contexts. Bracken‐Roche et al. suggest, for example, the need for mid‐level guidance between general guidance on responding to vulnerability and analyses conducted by research ethics boards.^18^ Furthermore, definitions of agency (and vulnerability) that fail to account for its fluctuation and dynamism throughout people's interactions with research studies perpetuate the longstanding issue in research ethics that consent still carries the major burden of being the primary (and sometimes only) site of ethical assessment and check on someone's autonomy and vulnerability.

Finally, beyond a need for greater uptake of existing feminist approaches to vulnerability, agency, and autonomy within research ethics guidance, practice, and research structures that influence accepted approaches to research, more attention is needed to what obligations arise and for whom at different stages of the research pathway in light of changes in thinking about these concepts.

To help ground this discourse on vulnerability and agency, and illustrate key implications for research ethics, we will share reflections on a vignette based on empirical research ethics work by NKosi, Seeley and colleagues in South Africa.^19^ This vignette comprises a composite of several real scenarios and is representative of the ethical challenges faced in this and similar contexts. Persons, institutions, and locations have been de‐identified.

*Research is being conducted in KwaZulu‐Natal on HIV prevention programmes amongst children and young people. Because of their age, research participants under 18 years old are considered vulnerable, and local ethical guidance and review processes require certain provisions to be put in place for their protection, such as consent from a legal parent or guardian, and a commitment to follow mandatory reporting of safeguarding concerns to child protective services. Researchers were not required by guidance/review processes to consider ways to recognise the autonomy and agency of their participants beyond putting in place an assent process to accompany consent from a legal parent/guardian. Noma, a 16‐year‐old girl from KwaZulu‐Natal, is approached to participate in the study. She expresses interest in joining and asks questions about it, but says she is unsure whether she can participate. She lives with her parents, older sister, and younger brother. Things are challenging at home, including owing to a low household income and a difficult relationship with her parents. She is close to her sister and would like her to come along to hear about the research and give consent. However, the study protocol won't allow for this, and she is told that a parent or legal guardian must give consent. She reluctantly raises this with her mother, and eventually, her mother gives consent. However, it becomes clear to the researchers that the mother has different motives for having her daughter participate. Her mother tells the researchers she hopes to use the research as a means to find out whether her daughter is sexually active and says she hopes that the researcher will use the research to ‘teach her daughter about appropriate sexual behaviours.’ Soon after, during a research visit, a researcher notices signs of physical violence on Noma and asks about the injuries. Noma discloses that she is being abused but doesn't want the researcher to report it for fear of backlash from her family and neighbours. Since the participant is underage, and according to the study's ethics protocol, researchers are required to involve the local child protection officer. The child protection officer reports the case to the local police who visit the girl's home. The girl subsequently drops out of the research study and is lost to follow‐up. The researchers later hear through word of mouth that the girl got into trouble with her parents; the community also reacted negatively towards the girl and her family. Beyond the police visiting the home, no further action was taken, and no other services were involved*.

This vignette demonstrates a number of shortcomings with current research ethics practice and guidance for research with populations considered vulnerable. First, there is the focus on protection from harm over support and recognition of agency in the strict requirements for consent from a legal guardian, despite this person not necessarily representing the child's best interests. Second, the approach to safeguarding excludes consideration or respect for Noma's own wishes or capacity to determine the best course of action. The case was reported to child protective services without Noma's consent or involvement, and whilst researchers were following ethical guidance on this matter, this response did not provide Noma with the support she required. Overall, there was little attention given to the factors and services contributing to and alleviating vulnerability and agency in Noma's life, and as such, the response to this ethical dilemma both failed to provide her with adequate support, whilst at the same time exacerbating her vulnerability in the face of community stigma.

With this vignette in mind, we turn now to consideration of core concepts and critiques of agency emerging from gender and development and childhood studies. We consider how core conceptual arguments around agency as relational, ambiguous, and multidimensional could improve practical research ethics and guidance when working with participants like Noma in similar socioeconomic contexts, referring to the vignette to illustrate.

## 3 AGENCY IN THE CONTEXT OF VULNERABILITY FROM CHILDHOOD STUDIES AND GENDER AND DEVELOPMENT DISCOURSE

During the late 20th century, there was significant discussion across disciplines about the influence of social and material structures on people's agency. The ‘structure‐agency debate’ dominated the discussion, deriving mainly from work by Giddens and Bourdieu (amongst others) on structuration and practice theory.^20^ By recognising the influence of social structure (including institutions of family, religion, politics, law, and economy) over individuals' opportunities, choices, and actions, debate moved away from starker understandings of ‘free will’ long embraced by some in Western philosophy. Nevertheless, much usage of agency within the social sciences is still underpinned by post‐enlightenment liberal ideas of people as individual social agents, taking purposive, rational actions.^21^ Indeed, with the advance of neoliberalism, agency has become increasingly individualised, so that actions of groups, organisations, and movements are often seen to derive from individual members, without recognition of collective efforts and of collective duties, such as those accounted for by Collins' in her work on group duties.^22^

In recent years in gender and development and childhood studies, efforts to challenge the association of vulnerability with passivity and victimhood have at times been criticised for supporting these individualistic accounts of agency, for overemphasising agency, and for equating agency with overt acts of resistance to oppression.^23^ In childhood studies, some suggest that the promotion of children as social actors (to counter their historic portrayal as passive and dependent) has now become so ubiquitous as to be an ‘almost taken‐for‐granted mantra.’^24^ There is concern that while children's agency has been acknowledged in a multitude of constrained circumstances, its limitations may not have been properly problematised.^25^ In international development, individualistic, neoliberal models for promoting agency and empowerment of women and girls have been critiqued for depicting them as, ‘idealised agents of development.’^26^ Adolescent girls in particular have been portrayed as key agents of change, with the potential to disrupt and challenge social norms and structures that have previously disempowered them.^27^

These idealised models of liberal autonomy have been criticised for perpetuating notions of independent social actors from the Global North, bearing little resemblance to the reality for girls (and others) in various contexts, whose opportunities, choices, and actions are enabled and restricted by social and material circumstances. Overemphasis on individuals’ agency risks ignoring or concealing the presence of diverse social and structural constraints in their lives.^28^ Equally, equating agency with individual acts of open resistance to injustice risks obfuscating the strength of power structures that constrain people in different ways.^29^ As such, research and interventions may leave underlying structural drivers of vulnerability unchanged, or create further harm for marginalised participants where changes provoke resentment or backlash amongst those who benefit from maintaining oppressive societal structures.

Within work on children's agency in low‐ and middle‐income countries, discourses from the Global North around individual human agency and rights have been criticised for, ‘continuing colonial imperialism and of introducing ideas antithetical to certain cultures and traditions.’^30^ The lack of fit between Global North accounts of autonomy and people's real capacities and situations has two implications: First, it means that accounts of obligations, responsibilities, and goals will not fairly track people's lives, making practical ethics guidance irrelevant or out of touch with real experience. Second, a focus on individual power and responsibility risks placing responsibility for action onto individuals, as opposed to highlighting the need for collective, institutional, and state responses.^31^ Interventions and policies underpinned by such understandings may be over‐optimistic, placing unrealistically high expectations on people's capacity to enact change for and by themselves.^32^

### 3.1 Relational agency

Similar to arguments from feminist ethicists for relational understandings of autonomy, such as Mackenzie and Stoljar,^33^ relational theorists in childhood studies argue that to better reflect real human interactions in context, agency and vulnerability must be understood from a relational perspective: seeing all people and things as interlinked and interdependent, and seeing agency and vulnerability, not as inherent traits of individual entities but arising from, shaped by, and reproduced through interactions between whole networks of different human and nonhuman actors.^34^ Under these relational approaches, there is a strong rejection of agency as the property of a subject, or in opposition to constraining or enabling social structures; it is said to derive instead from relations between people, institutions, structures, and processes.^35^

Historically, tension existed between recognising someone's agency and, as described by leading theorists on children's agency, Tisdall and Punch, ‘acknowledging their position of vulnerability in a context of extreme structural constraints.’^36^ A relational approach overcomes this binary; instead of depicting vulnerability and agency as oppositional or inherent, they are seen arising, often simultaneously, from relationships. Increasingly, relational ethicists and philosophers argue against the traditional, oppositional agency–vulnerability dichotomy, with authors instead promoting their mutually constituted nature.^37^ Butler argues for recognition, for example, of ways that vulnerability can be constitutive of agency and resistance, such as where people mobilise their bodily vulnerability in nonviolent protest against social injustices and state brutality.^38^ Research on children's agency helps demonstrate in practice how expressions of agency can arise through experiences of vulnerability, and vice versa, so that individuals experience manifestations of both simultaneously. Mizen and Ofosu‐Kusi's paper on migrant child workers in Ghana, for example, demonstrates how causes of children's vulnerability, such as poverty and mistreatment, also play a contributory role in their experiences of agency, in this instance in carefully deliberated and enacted decisions to leave home.^39^

The body of work on women and children's agency supports feminist ethicists and philosophers advocating for relational approaches to ethical obligations (e.g., Rogers, Mackenzie, Luna), urging research ethics to move beyond dualist framings of agency and structure, and definitions of vulnerability and agency as inherent traits. Relational approaches help dissolve other dichotomies—including those posing vulnerability and agency, or victim and agent, as oppositional. Looking beyond these binaries can reveal often hidden, multifaceted aspects of agency, even within highly constrained and challenging circumstances, such as we see with Noma in her interest in joining the research study.^40^ Concurrently, discourse on women and children's agency warns against an over‐attribution of agency. If we overlook the apparent risks to Noma's wellbeing, for instance, including signs of abuse, we could miss core ethical concerns in her situation. Conceptualising agency or vulnerability as inherent demarcates some individuals as ‘having agency’ and others as having none. With this comes the risk of either inadequately protective or overly paternalistic responses. The parallel discourses on relational approaches to vulnerability and agency in ethics and gender and development and childhood studies both help to reveal the possibility of supporting Noma's agency whilst at the same time recognising her position of vulnerability. In practice, this could mean pushing back on the need for a legal guardian to consent, recognising either her own capacity to make this decision for herself, or recognising the relationships she values, such as those of her older sister or potentially even someone outside of the family.

When agency and vulnerability are seen to arise from relationships, research ethics is not responding to traits of individuals but to dynamic and shifting interconnections between people, structures, and processes, requiring responses that take these realities into account. Arguments from feminist ethicists for more relational approaches in bioethics have gone a long way to increase how researchers and clinicians see the importance of interpersonal relationships. But research ethics must still go further, to recognise how vulnerabilities and agency arise from other forms of relationship in the research setting. Accounts of women and children's agency from gender and development and childhood studies advance the aspects of relational theory that emphasise how agency and vulnerability arise from relations between people, institutions, and social structures, as well as between individual participants, household members, and researchers. In the vignette, for example, we see how critical it is for the researchers to understand how support systems like child protective services work ‐ particularly how to navigate an inflexible and overwhelmed system so as to avoid causing further harm to Noma. Relational approaches indicate that researchers and research guidance should look beyond these ‘close‐in’ relationships between participants and households, towards relationship frameworks that connect participants, households, researchers, research institutions, and broader systems of health inequality and injustice. Beyond lending weight to existing arguments for the adoption of relational approaches to both vulnerability and agency, childhood studies and gender and development scholarship has also provided accounts of agency as ambiguous and multifaceted. We suggest that these accounts can further enhance research ethics practice and guidance in research with vulnerable populations.

### 3.2 Agency as ambiguous and value‐laden

Bordonaro and Payne describe ‘ambiguous agency’ as expressions of agency that go against normative ideas of what constitutes good or appropriate actions.^41^ Agency is often seen as wholly positive, but expressions of agency can have both positive and negative implications; agency can even be self‐destructive in the short or long term.^42^ It is important to recognise that definitions of agency are value‐laden. Attributions of agency can mask underlying moral judgements about what society categorises as ‘good’ or ‘bad’ actions and choices. For example, how we understand children's agency is based on how we understand childhood itself, what we see as appropriate or inappropriate actions and responsibilities for children, conceptions of which vary across time and context. Consider a child leaving school to work to help support their family. Through analysis of child work in Ethiopia, Boyden highlights the possibility of both negative and positive implications—negative impacts on formal education, but potential opportunities to develop pro‐social skills, transition to adulthood, and contribute to household financial security.^43^ Pells et al. describe ambiguous forms of agency amongst women experiencing intimate partner violence in Vietnam. While not openly challenging violence, women still found agential ways within their current situations to meet the needs of their children and families. Pells et al. ask that we not disregard more indirect, hidden, ambiguous expressions of agency, simply because they do not conform to Westernised, positive definitions of the concept.^44^

The concept of ambiguous agency challenges our preconceptions as to what kinds of agency research ethics should be fostering, especially in circumstances of vulnerability and constraint. It contradicts our concern for Noma's protection, for instance, to ignore signs of abuse and do nothing to respond, despite it being against Noma's wishes (and potentially best interests) to follow reporting guidelines. Work on ambiguous agency underscores the importance of avoiding assumptions about what kind of responses and supports might be beneficial for participants experiencing vulnerability. It confirms the need to evaluate the presence and quality of participants' social support, including potentially harmful social relations. For example, much about how researchers ought best to respond to dilemmas in the vignette turns on appreciating Noma's complex relationship with her mother, other family members, and neighbours.

Work on the ambiguous, value‐laden nature of agency highlights that if we want research to help foster agency, we need to be wary about what values and assumptions underpin our interpretations of what counts as agency. This points again to the importance of strengthening support systems and structures in the broader study context so that participants have real opportunities for support that they can then draw on or not, depending on what they determine best for themselves at the time. There are few requirements currently in research ethics guidance or governance and review processes to really assess or understand the factors that contribute to and detract from participants’ agency in the research context, and yet work on vulnerability has long highlighted the possibility that familiarity with study context and the development of long‐term relationships with local communities can be beneficial for mitigating participants' vulnerability.

In Noma's case, this underscores the importance of equipping participants properly from the start with the information they need regarding study safeguarding mechanisms and reporting etc., in order for them to interact with the study in accordance with their wishes and retain influence over how their situation is responded to (or not), but it also highlights the importance of embedding the study within a network of support upon which participants can choose (or not) to draw. The likelihood of encountering instances of interpersonal and gender‐based violence can often be pre‐empted and ways to support participants’ agency without diminishing or invalidating it put in place from the start. Many researchers already establish processes for participants at the beginning of studies, such as presentations from and introductions to local support services, or referral letters issued automatically to every participant as opposed to by individual request. Both these measures aim to assist participants to access support on their own terms, without having to disclose abuse intentionally or unintentionally to study staff (which in Noma's case appeared to impact negatively on her agency).

### 3.3 Multiplicity of agency

Whilst there have been arguments made by feminist theorists for research ethics to adopt more situated, context‐specific, and diverse accounts of vulnerability, work on children and women's agency argues correspondingly that there is no one ‘type’ of agency, but that multiple types manifest differently, often simultaneously, depending on the person, place, or time.^45^ Indeed, universalised approaches in international development interventions and policies have been criticised for not recognising agency as context‐specific, situated, dynamic, and distributed.^46^ In synthesising research on agency amongst marginalised women, Campbell and Mannell highlight four ‘dimensions’ of how agency is distributed—across time, space, social networks, and along a ‘continuum of activism.’^47^ They challenge accounts of agency limited to single actions by individual people at discrete moments, and instead emphasise the temporality of agency, so that individuals are seen as neither victims nor agents, but as people experiencing fluctuating agency throughout their daily lives. They assert that agency is dependent on the presence and quality of material and social support networks, as well as on space—both geographically and in terms of positionality within local‐national‐global power structures. Lastly, they argue for greater recognition that agency is distributed across a continuum of actions so that not only those individual acts of overt resistance are recognised as agential, but also more subtle or hidden acts of persistence and coping, as well as collective responses. This latter view is supported by Kawarazuka et al.′s work on creative agency amongst women in coastal Kenya. Through analysis of negotiations around food provisioning, they highlight the women's ability to act creatively and strategically to meet their own interests (imbricated with those of their families and wider social networks), working within patriarchal structures as opposed to radically challenging them.^48^ This supports McNay's earlier work on creative agency, which claimed that agency could be creative or productive in the context of constraint.^49^ As with calls from feminist ethicists to differentiate types of vulnerability, recognition of a broader spectrum of agency is needed within research ethics guidance and practice in order to better understand and support those experiencing vulnerability.^50^

The accounts of agency outlined here highlight the need for research ethics practice to be informed by people's self‐perceptions of agency in their lives.^51^ Indeed, Payne explains how studies of child‐headed households in the global South often contrast children's amazing resilience with their inherent vulnerability, highlighting remarkable actions in response to extreme difficulty. She argues this is inconsistent with how children characterise their own experiences of agency as part of everyday life, terming this ‘everyday agency.’ This has implications for how agency should be characterised in research ethics practice and guidance going forward, suggesting accounts and responses should reflect people's own perspectives of their lives and agency, instead of ascribing meaning top‐down.^52^

This reinforces existing calls for ongoing, collaborative, participatory, ethical encounters with participants, but with additional requirements to ensure aims to ‘foster agency’ are fit for purpose in reflecting real daily lives, needs, and perspectives of those involved. In Noma's case, a greater appreciation for people's self‐characterisations of agency, vulnerability, and the kinds of support they need might encourage the researchers to listen carefully to Noma, and engage more with participants, community members, and other stakeholders on how to improve responses to domestic violence more widely. A local young people's advisory group (YPAG) could be established, where girls like Noma could discuss the research and feedback on the consent process, the research questions, and how the research should support young people during and after the study. In working out what should be done to support her, it is clear Noma can and should be engaged directly, perhaps discussing researchers' ethical and legal obligations to contact child protective services with safeguarding concerns, and having her guide how her situation is responded to. Under certain circumstances, there may be laws around mandatory reporting, but there may be different ways to navigate within these, to encourage participants to speak to someone they trust or to think together about other sources of support.

Recognition that agency and vulnerability are distributed differently across time, social networks, and space, with support fluctuating and varying in people's lives during their interaction with research, further indicates that ethical reflections and responses need to be ongoing throughout the research pathway, rather than reserved for discrete points—such as ethics review and informed consent. There is a need for ongoing engagement with participants and other stakeholders throughout studies, not just in design and priority‐setting, but during implementation and post‐study, enabling the study team to be responsive to needs as they fluctuate. However, there is also a need for ongoing ethics support for researchers, clinicians, and other frontline staff upon whom the burden of moral decision‐making often rests during studies. In Noma's case, frontline researchers may have benefitted from greater opportunity and encouragement to discuss ethical challenges with colleagues or other stakeholders, who could offer insight, potential responses, as well as a sense of shared ethical responsibility.

## 4 KEY IMPLICATIONS FOR RESEARCH ETHICS RESPONSIBILITIES

First, through reinforcing feminist ethicists' calls for a relational approach to agency and vulnerability in the research context, the accounts above indicate that research institutions' responsibilities might not be limited to individual participants but may extend to supporting or advocating for development of proper support networks and synchronicity across institutions. A relational approach may even give rise to obligations to address structural drivers of inequality, as well as to support more collective forms of agency, potentially shifting how research has traditionally responded to the needs of individual participants.

Second, in recognising agency as ambiguous and value‐laden, obligations also arise for research ethics guidance and practice to allow participants to determine what agency means to them, rather than basing judgements solely on externally determined criteria. This has long been recognised by anthropologists supporting the emic view—to understand phenomena from the perspective of ‘cultural insiders.’^53^ Feminist theorists have also argued for some time that definitions of vulnerability in the research ethics contexts be informed by participants' self‐perceptions. Work on women and children's agency emphasises the need for equivalent attention to self‐perceptions of agency; if the concept of agency is externally defined and simply translated to other contexts, multiple ways in which people employ agency, including how they may strategically navigate and utilise the structures and systems they are familiar with and yet constrain them, maybe missed or downplayed.

Third, multidimensional accounts of agency signify that ethical reflection and response should be expanded across the research pathway, rather than being limited to informed consent, providing ethical support for both participants and research staff. Whilst this insight is not new in the theoretical literature, research ethics practice still lags behind. Even arguments for relational approaches to research ethics have tended to concentrate on expanding consideration of who is involved in decision‐making regarding informed consent procedures. What is needed is support for, as well as the commitment of time and resources to, ongoing research ethics support throughout the research cycle. Elsewhere we have developed tools to support a more dynamic approach to research ethics support.^54^

Finally, discussing implications for research obligations of a relational, multi‐faceted, dynamic, and highly contextual understanding of agency also raises questions about where the limits of such responsibilities lie, particularly with regard to addressing and transforming structural sources of injustice. We are not suggesting that research should bear unbounded responsibility for responding to all relevant aspects of agency and vulnerability in a given situation, which would be unrealistic resource‐wise and potentially inappropriate from a positionality perspective. Nevertheless, there may be obligations which fall more reasonably within the remit of research—including regarding advocacy to shift the priorities and practices of power holders, as well as strengthening collaborations with and capacities of those better‐placed to respond directly to issues revealed during the research. There may also be responsibilities to shine a light inward on practices and structures of research institutions themselves, as these pertain to the agency and vulnerability of research populations. Who decides, who manages, and who carries out the research matter for institutions’ abilities to fairly engage with and make a sustained difference for populations, and hence reflection on institutional as well as societal‐wide power structures and imbalances may be required.

Rather than a predetermined set of responsibilities, we recommend an overarching responsibility to pay greater attention to structural factors which help shape vulnerability and agency, as well as to take a more open, flexible, and active approach to understanding and responding to issues of vulnerability and agency in specific research contexts. This latter point is particularly pertinent to research conducted across multiple settings, which may need to account for more differences than multi‐site research tools, randomisation criteria, etc., have previously assumed.

## 5 CONCLUSION

As the importance of fostering participants' agency has become more prominent in research ethics guidance and discourse, we have argued that there is much still to be embedded in guidance and practice from work by feminist relational ethicists and philosophers, as well as to be learned from critiques of agency within childhood studies and gender and development discourses. These corresponding bodies of work reinforce calls to move beyond idealised, individualistic models of autonomy and agency, and to recognise and respond to the interdependency of people, institutions, and structures. This requires a shift in how we understand vulnerability and agency, not only in guidance but also within research structures and institutions that currently determine which approaches to research ethics become accepted practice. Extensive work demonstrates the inapplicability of individualised characterisations of agency from the Global North to other settings internationally, and indeed to many settings from which they initially derived, calling for more situated and contextually grounded definitions that recognise the heterogeneity of agency (and vulnerability) people experience. Moreover, in highlighting its ambiguity, critiques of women and children's agency in under‐resourced settings call attention to the normative work being done by the concept that often remains hidden. If research and interventions are to achieve their goal of supporting agency in the context of vulnerability, exposing definitions (and the moral judgements which underpin them) to scrutiny and reflection, and being flexible enough to respond to changes in people's agency and circumstances, will be crucial. Fundamental to these aims, it remains critical that we enable local communities and participants to inform definitions of agency and shape responses based on their own experiences and self‐perceptions.

## 6 CONFLICT OF INTEREST STATEMENT

The authors declare no conflicts of interest.

## Footnotes

1 Jennings, B. (2016). Reconceptualizing autonomy: A relational turn in bioethics. *Hastings Center Report*, *46*(3), 11–16.

2 Mackenzie, C., Rogers, W., & Dodds, S. (Eds.). (2014). *Vulnerability: New essays in ethics and feminist philosophy*. Oxford University Press; Luna, F. (2009). Elucidating the concept of vulnerability: Layers not labels. *International Journal of Feminist Approaches to Bioethics*, *2*(1), 121–139.

3 Sherwin, S. (2008). Whither bioethics? How feminism can help reorient bioethics. *International Journal of Feminist Approaches to Bioethics*, *1*(1), 7–27, p. 12.

4 Scambler, G. (2019). Dimensions of vulnerability salient for health: A sociological approach. *Society, Health & Vulnerability*, *10*(1), 1557467; Mackenzie, C., et al., op. cit. note 2.

5 Tripathi, T., & Mishra, N.K. (2017). Fuzziness in conceptualisation of women's empowerment, access to resources and autonomy. *Journal of Social and Economic Development*, *19*(1), 60–82; Kagitcibasi, C. (2005). Autonomy and relatedness in cultural context. *Journal of Cross‐Cultural Psychology*, *36*(4), 403–422.

6 Bracken‐Roche, D., Bell, E., & Racine, E. (2016). The ‘vulnerability’ of psychiatric research participants: Why this research ethics concept needs to be revisited. *Canadian Journal of Psychiatry*, *61*(6), 335–339; Mackenzie, C., et al., op. cit. note 2.

7 Luna, F. (2022). Vulnerability and feminist bioethics. In W. A. Rogers, J. L. Scully, S. M. Carter, V. A. Entwistle, & C. Mills (Eds), *The Routledge handbook of feminist bioethics* (pp. 96–109). New York: Routledge; Mackenzie, C., et al., op. cit. note 2; Lange, M. M., Rogers, W., & Dodds, S. (2013). Vulnerability in research ethics: A way forward. *Bioethics*, *27*(6), 333–340; Mackenzie, C., McDowell, C., & Pittaway, E. (2007). Beyond ‘do no harm’: The challenge of constructing ethical relationships in refugee research. *Journal of Refugee Studies*, *20*(2), 299–319.

8 Hugman, R., Pittaway, E., & Bartolomei, L. (2011). When ‘do no harm’ is not enough: The ethics of research with refugees and other vulnerable groups. *British Journal of Social Work*, *41*(7), 1271–1287; Lange, M. M., et al., op. cit. note 7, pp. 333–340.

9 Ibid: 337.

10 Farsides, B., Brierley, J., Coyne, I., Davis, E., Fovargue, S., Gill, R., Jackson, R., Marsh, V., Molyneux, S., Sammons, H., Sheehan, M., Tansey, S., Taylor, M., & Young, B. (2015). *Children and clinical research: Ethical issues*. Nuffield Council on Bioethics; Hugman, R., et al., op. cit. note 8; Thomas, N., & O'Kane, C. (1998). The ethics of participatory research with children. *Children & Society*, *12*(5), 336–348.

11 Hahn, D. L., Hoffmann, A. E., Felzien, M., LeMaster, J. W., Xu, J., & Fagnan, L. J. (2017). Tokenism in patient engagement. *Family Practice*, *34*(3), 290–295; Absalom, E., Chambers, R., Francis, S., Gueye, B., Guijt, I., Joseph, S., Johnson, D.,  Kabutha, C., Khan, M. R., Leurs, R., Mascarenhas, J., Norrish, P., Pimbert, M., Pretty, J., Samaranyake, M., Scoones, I., Shah, M. K., Shah, P., Tamang, D., Thompson, J., Tym, G., & Welbourn, A. (2001). Sharing our concerns and looking to the future. *PLA Notes*, 22, 5–10.

12 Luna, op. cit. note 7.

13 Bracken‐Roche, D., Bell, E., Macdonald, M.E., & Racine, E. (2017). The concept of ‘vulnerability’ in research ethics: In‐depth analysis of policies and guidelines. *Health Research Policy and Systems*, *15*(1), 1–18; Dove, E. S., Kelly, S. E., Lucivero, F., Machirori, M., Dheensa, S., & Prainsack, B. (2017). Beyond individualism: Is there a place for relational autonomy in clinical practice and research? *Clinical Ethics*, *12*(3), 150–165; Bracken‐Roche, D., et al., op. cit. note 6.

14 Kelley, M. C., Brazg, T., Wilfond, B. S., Lengua, L. J., Rivin, B. E., Martin‐Herz, S. P., & Diekema, D. S. (2016). Ethical challenges in research with orphans and vulnerable children: A qualitative study of researcher experiences. *International Health, 8*(3), 187–196; Singh, J. A., Karim, S. S. A., Karim, Q. A., Mlisana, K., Williamson, C., Gray, C., Govender, M., & Gray, A. (2006). Enrolling Adolescents in Research on HIV and Other Sensitive Issues: Lessons from South Africa. *PLOS Medicine, 3*(7), e180.

15 Council for International Organizations of Medical Sciences (CIOMS). (2016). *International ethical guidelines for health‐related research involving humans*. CIOMS.

16 Khirikoekkong, N., Jatupornpimol, N., Nosten, S., Asarath, S. A., Hanboonkunupakarn, B., McGready, R., Nosten, F., Roest, J., Parker, M., Kelley, M., & Cheah, P. Y. (2020). Research ethics in context: Understanding the vulnerabilities, agency and resourcefulness of research participants living along the Thai–Myanmar border. *International Health*, *12*(6), 551–559; Nkosi, B., Seeley, J., Chimbindi, N., Zuma, T., Kelley, M., & Shahmanesh, M. (2020). Managing ancillary care in resource‐constrained settings: Dilemmas faced by frontline HIV prevention researchers in a rural area in South Africa. *International Health*, *12*(6), 543–550; Zakayo, S. M., Njeru, R. W., Sanga, G., Kimani, M. N., Charo, A., Muraya, K., Sarma, H., Uddin, F., Berkley, J. A., Walson, J. L., Kelley, M., Marsh, V., … Molyneux, S. (2020). Vulnerability and agency across treatment‐seeking journeys for acutely ill children: How family members navigate complex healthcare before, during and after hospitalisation in a rural Kenyan setting. *International Journal for Equity in Health*, *19*(1), 1–17; Bracken‐Roche, D., et al., op. cit. note 13.

17 Campbell, C., & Mannell, J. (2016). Conceptualising the agency of highly marginalised women: Intimate partner violence in extreme settings. *Global Public Health*, *11*(1–2), 1–16; Ahearn, L. M. (2001). Language and agency. *Annual Review of Anthropology*, *30*(1), 109–137, p. 130.

18 Bracken‐Roche, D., et al., op. cit. note 12.

19 NKosi, B., Seeley, J., Chimbindi, N., Zuma, T., Kelley, M., & Shahmanesh, M. (2022). Putting research ethics in context: Rethinking vulnerability and agency within a research ethics case study on HIV prevention for young girls in South Africa. *SSM‐Qualitative Research in Health, 2*, 100081.

20 Giddens, A. (1984). *The constitution of society: Outline of the theory of structuration*. University of California Press; Bourdieu, P. (1977). *Outline of a theory of practice*. Cambridge University Press; Taylor, C. (1989). *Sources of the self: The making of the modern identity*. Harvard University Press.

21 Lerch, J., Bromley, P., Ramirez, F.O., & Meyer, J. W. (2017). The rise of individual agency in conceptions of society: Textbooks worldwide, 1950–2011. *International Sociology*, *32*(1), 38–60; Asad, T. (2000). Agency and pain: An exploration. *Culture and Religion*, *1*(1), 29–60.

22 Lerch, J., et al., op. cit. note 21, p. 39; Collins, S. (2019). Collectives and their duties. In S. Collins (Ed.), *Group duties: Their existence and their implications for individuals* (pp. 153–180). Oxford University Press.

23 Madhok, S., Phillips, A., & Wilson, K. (2013). *Gender, agency and coercion*. Palgrave Macmillan.

24 Punch, S. (2016). Exploring children's agency across majority and minority world contexts. In F. Esser, M. S. Baader, T. Betz, & B. Hungerland (Eds.), *Reconceptualising agency and childhood: New perspectives in childhood studies* (pp. 183–196). Routledge, p. 184; Tisdall, E. K. M., & Punch, S. (2012). Not so ‘new’? Looking critically at childhood studies. *Children's Geographies*, *10*(3), 249–264.

25 Ibid.

26 Wilson, K. (2015). Towards a radical re‐appropriation: Gender, development and neoliberal feminism. *Development and Change*, *46*(4), 803–832, p. 818.

27 Madhok, S., et al., op. cit. note 23.

28 Campbell & Mannell, op. cit. note 17; Madhok, S., et al., op. cit. note 23.

29 Abebe, T. (2019). Reconceptualising children's agency as continuum and interdependence. *Social Sciences*, *8*(3), 81; Madhok, S., et al., op. cit. note 23; Asad, op. cit. note 21.

30 Tisdall & Punch, op. cit. note 24, p. 250.

31 Abebe, op. cit. note 29; Madhok, S., et al., op. cit. note 23; Asad, op. cit. note 21.

32 Wilson, op. cit. note 26.

33 Stoljar, N., & Mackenzie, C. (2022). Relational autonomy in feminist bioethics. In W. A. Rogers, J. L. Scully, S. M. Carter, V. A. Entwistle, & C. Mills (Eds.), *The Routledge handbook of feminist bioethics* (pp. 71–83). Routledge.

34 Esser, F. (2016). Neither ‘thick’ nor ‘thin’: Reconceptualising agency and childhood relationally. In F. Esser, M. S. Baader, T. Betz, & B. Hungerland (Eds.), *Reconceptualising agency and childhood: New perspectives in childhood studies* (pp. 48–60). Routledge; Oswell, D. (2016). Re‐aligning children's agency and re‐socialising children in Childhood Studies. In F. Esser, M. S. Baader, T. Betz, & B. Hungerland (Eds.), *Reconceptualising agency and childhood: New perspectives in childhood studies*. Routledge.

35 Esser, op. cit. note 34.

36 Tisdall & Punch, op. cit. note 24, p. 256.

37 Abebe, op. cit. note 29.

38 Butler, J. (2016). Rethinking vulnerability and resistance. In J. Butler, Z. Gambetti, & L. Sabsay (Eds.), *Vulnerability in resistance* (pp. 12–27). Duke University Press.

39 Mizen, P., & Ofosu‐Kusi, Y. (2013). Agency as vulnerability: Accounting for children's movement to streets of Accra. *The Sociological Review*, *61*(2), 363–382.

40 Campbell & Mannell, op. cit. note 17, p. 1.

41 Bordonaro, L. I., & Payne, R. (2012). Ambiguous agency: Critical perspectives on social interventions with children and youth in Africa. *Children's Geographies*, *10*(4), 365–372.

42 Gigengack, R. (2008). Critical omissions: How street children studies can address self‐destructive agency. In P. Christensen & A. James (Eds.), *Research with children: Perspectives and practices* (pp. 205–219). Routledge.

43 Boyden, J. (2009). Risk and capability in the context of adversity: Children's contributions to household livelihoods in Ethiopia. *Children, Youth and Environments*, *19*(2), 111–137, p. 130.

44 Pells, K., Wilson, E., & Thi Thu Hang, N. (2016). Negotiating agency in cases of intimate partner violence in Vietnam. *Global Public Health*, *11*(1–2), 34–47, p. 41.

45 Ahearn, op. cit. note 17; MacLeod, A. E. (1992). Hegemonic relations and gender resistance: The new veiling as accommodating protest in Cairo. *Signs: Journal of Women in Culture and Society*, *17*(3), 533–557.

46 Abebe, op. cit. note 29, p. 11; Mohanty, C.T. (2013). Transnational feminist crossings: On neoliberalism and radical critique. *Signs: Journal of Women in Culture and Society*, *38*(4), 967–991, p. 967.

47 Campbell & Mannell, op. cit. note 17, p. 13.

48 Kawarazuka, N., Locke, C., & Seeley, J. (2019). Women bargaining with patriarchy in coastal Kenya: Contradictions, creative agency and food provisioning. *Gender, Place & Culture*, *26*(3), 384–404, p. 399.

49 McNay, L. (2000). *Gender and agency: Reconfiguring the subject in feminist and social theory* (p. 5). Polity Press.

50 Campbell & Mannell, op. cit. note 17.

51 Ibid; Payne, R. (2012). ‘Extraordinary survivors’ or ‘ordinary lives’? Embracing ‘everyday agency’ in social interventions with child‐headed households in Zambia. *Children's Geographies*, *10*(4), 399–411.

52 Ibid: 400.

53 Morris, M. W., Leung, K., Ames, D., & Lickel, B. (1999). Views from inside and outside: Integrating emic and etic insights about culture and justice judgment. *The Academy of Management Review*, *24*(4), 781–796, p. 783.

54 Molyneux, S., Sukhtankar, P., Thitiri, J., Njeru, R., Muraya, K., Sanga, G., Walson, J. L., Berkley, J., Kelley, M., & Marsh, V. (2021). Model for developing context‐sensitive responses to vulnerability in research: Managing ethical dilemmas faced by frontline research staff in Kenya. *BMJ Global Health*, *6*(7), e004937.

